# Anticancer Activity and Mechanisms of Action of New Chimeric EGFR/HDAC-Inhibitors

**DOI:** 10.3390/ijms22168432

**Published:** 2021-08-05

**Authors:** Nils Goehringer, Bernhard Biersack, Yayi Peng, Rainer Schobert, Marco Herling, Andi Ma, Bianca Nitzsche, Michael Höpfner

**Affiliations:** 1 Institute of Physiology, Charité-Universitätsmedizin Berlin, Charitéplatz 1, 10117 Berlin, Germany; nils.goehringer@charite.de (N.G.); andi.ma@charite.de (A.M.); 2 Organic Chemistry 1, University of Bayreuth, Universitätsstraße 30, 95440 Bayreuth, Germany; bernhard.biersack@yahoo.com (B.B.); rainer.schobert@uni-bayreuth.de (R.S.); 3 Laboratory of Lymphocyte Signaling and Oncoproteome, University Hospital Cologne, Weyertal 115c, 50931 Cologne, Germany; yayipengmed@163.com (Y.P.); marco.herling@medizin.uni-leipzig.de (M.H.); 4 Clinic and Polyclinic for Hematology, Cell Therapy and Hemostaseology, Liebigstraße 22, House 7, 04103 Leipzig, Germany

**Keywords:** chimeric inhibitor, anticancer drugs, solid cancer, epidermal growth factor (EGFR), histone deacetylase (HDAC), anti-angiogenesis, prostate cancer, hepatoma, leukemia, lymphoma

## Abstract

New chimeric inhibitors targeting the epidermal growth factor (EGFR) and histone deacetylases (HDACs) were synthesized and tested for antineoplastic efficiency in solid cancer (prostate and hepatocellular carcinoma) and leukemia/lymphoma cell models. The most promising compounds, 3BrQuin-SAHA and 3ClQuin-SAHA, showed strong inhibition of tumor cell growth at one-digit micromolar concentrations with IC_50_ values similar to or lower than those of clinically established reference compounds SAHA and gefitinib. Target-specific EGFR and HDAC inhibition was demonstrated in cell-free kinase assays and Western blot analyses, while unspecific cytotoxic effects could not be observed in LDH release measurements. Proapoptotic formation of reactive oxygen species and caspase-3 activity induction in PCa and HCC cell lines DU145 and Hep-G2 seem to be further aspects of the modes of action. Antiangiogenic potency was recognized after applying the chimeric inhibitors on strongly vascularized chorioallantoic membranes of fertilized chicken eggs (CAM assay). The novel combination of two drug pharmacophores against the EGFR and HDACs in one single molecule was shown to have pronounced antineoplastic effects on tumor growth in both solid and leukemia/lymphoma cell models. The promising results merit further investigations to further decipher the underlying modes of action of the novel chimeric inhibitors and their suitability for new clinical approaches in tumor treatment.

## 1. Introduction

In the last decades, a plethora of novel approaches for the treatment of advanced cancers has been developed. Among them, the concept of co-inhibiting distinct cellular tumor targets by so-called “chimeric inhibitors,” which merge two drug pharmacophores into a single molecule, has become a promising approach [1]. Attacking the tumor in dual or multiple ways is thought to hamper the occurrence or activation of tumor escape mechanisms known for single-mode chemotherapeutics and may help to diminish chemoresistance [2]. Compared with combination therapies, where different active compounds are given simultaneously or sequentially to address distinct cellular targets, the use of chimeric compounds offers several advantages and circumvents problems typical of combination therapies, such as different drug solubilities or physical incompatibilities which may lead to precipitation or drug inactivation. In addition, the risk of drug–drug interactions or the occurrence of concomitant adverse or unwanted side effects, which often require a complex dose adjustment, can be avoided when using chimeric inhibitors instead [1,2,3].

In the development of novel chimeric inhibitors, the hybridization of histone deacetylase inhibitors with receptor tyrosine kinase inhibitory pharmacophores has emerged as a particularly promising anticancer approach [4]. Histone deacetylases (HDACs), which are overexpressed in various cancers, are epigenetic regulators of chromatin condensation and decondensation. They have a strong impact on cancer cell proliferation, spreading, and metastasis. This renders HDAC inhibitors a promising novel class of compounds for targeted cancer therapy. Some HDAC inhibitors, such as the hydroxamic acid derivatives vorinostat or panobinostat, are already approved for the treatment of hematologic cancer diseases and are currently under intensive investigation for their use in solid tumors [5]. There are also drawbacks of the clinical application of single HDAC inhibitors, such as intrinsic or acquired drug resistance [4]. These may be overcome by merging an HDAC-inhibitory pharmacophore with another tumor-relevant inhibitor. In this respect, a pharmacophore directed against the epidermal growth factor receptor (EGFR) may be a promising hybridization partner, as the EGFR is also overexpressed and/or overactive in various solid tumors, including hepatocellular and prostate cancer [6,7]. Small molecule inhibitors of the EGFR and its tyrosine kinase activity, such as gefitinib, erlotinib, or lapatinib, are already clinically approved for the treatment of solid cancers [8,9].

In this paper, we report on the synthesis and biological evaluation of novel chimeric inhibitors which consist of newly designed tyrphostin derivates functioning as epidermal growth factor inhibitors coupled with an HDAC-targeting hydroxamic acid moiety. HCC and PCa cell models were chosen as representatives of solid cancers and concomitant studies with lymphoma and leukemia cells were performed to underline the inhibitors relevance also for hematologic cancer diseases, thus indicating a broad spectrum of applicability. 

## 2. Results

### 2.1. Chemistry 

**Scheme 1:**
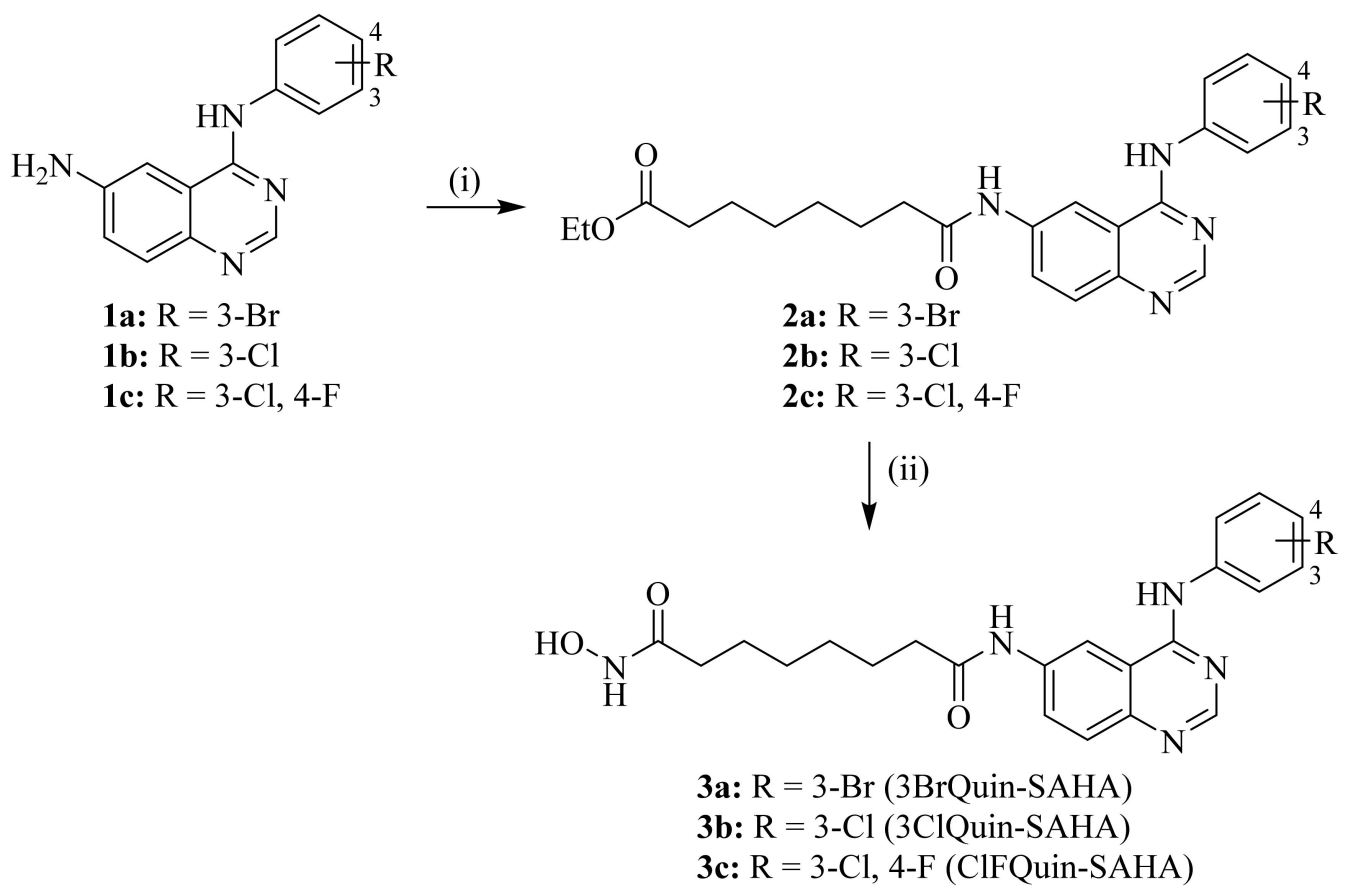
Reagents and conditions: (i) 1,8-dioctanoic acid mono ethyl ester, EDCI, DMAP, Et_3_N, CH_2_Cl_2_, r.t., 24 h, 39–47%; (ii) 50% aq. HO-NH_2_, NaOH, CH_2_Cl_2_/MeOH, r.t., 1 h, 43–64%.

First, 1,8-Dioctanoic acid mono ethyl ester was reacted with the corresponding 6-aminoquinolines 1a–c and 1-ethyl-3-(3-dimethylaminopropyl)carbodiimide (EDCI) to afford ester precursors 2a–c (Scheme 1). Then, target compounds 3a–c were obtained from the reaction of 2a–c with aqueous hydroxylamine under basic conditions. Compounds 3a–c have a chimeric structure composed of an EGFR-targeting anilinoquinazoline moiety similar to approved EGFR inhibitors, such as gefitinib, and an oxoalkylhydroxamic acid appendage related to clinically applied HDAC inhibitors, such as SAHA, designed to interact with the Zn ions of the HDAC active sites.

### 2.2. Biological Evaluation

#### 2.2.1. Antiproliferative Effects

**Figure 1:**
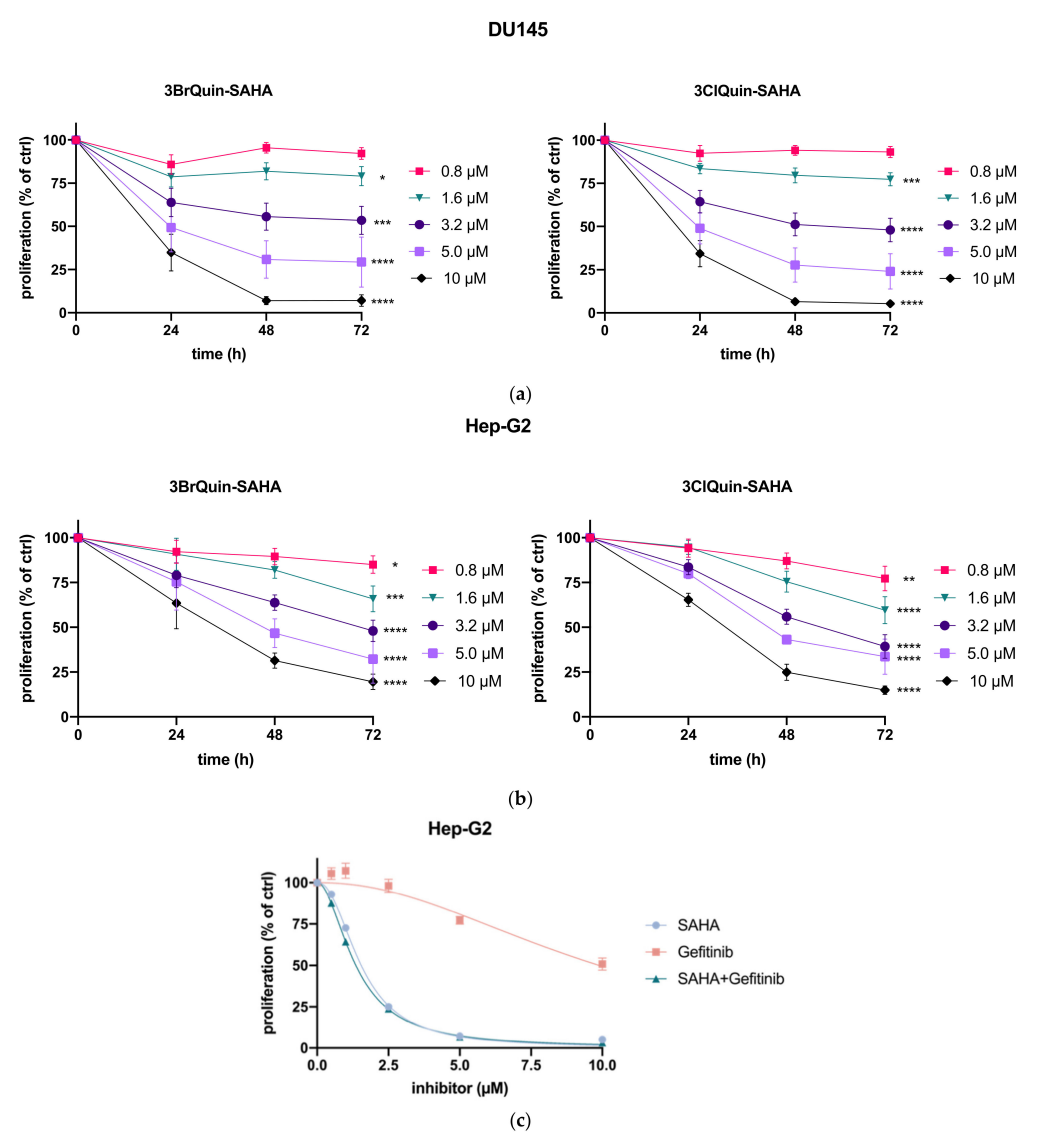

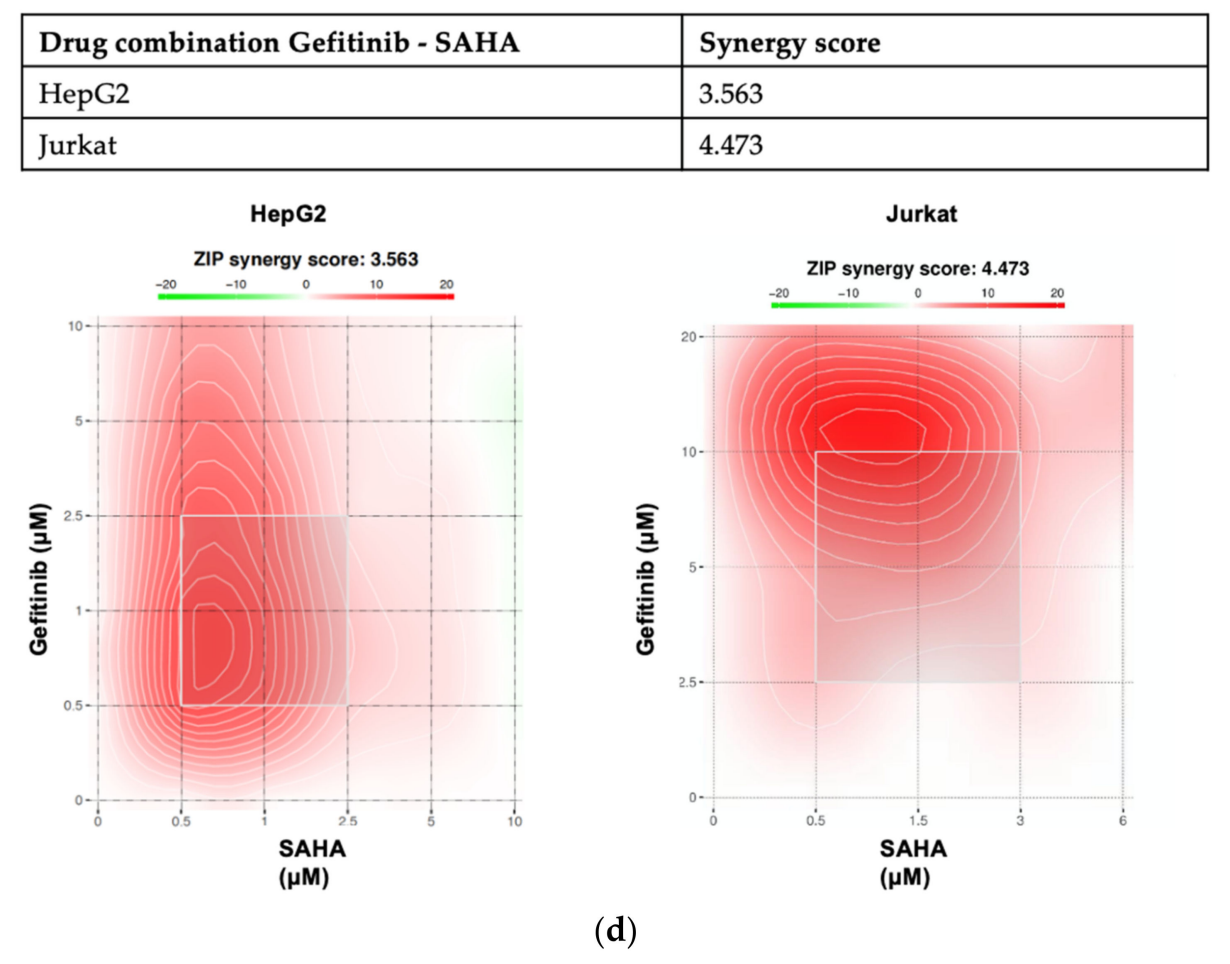
Growth-inhibitory effects of 3BrQuin-SAHA and 3ClQuin-SAHA in (**a**) PCa and (**b**) HCC cells. Time- and dose-dependent growth inhibition is given in percent, relative to untreated control cells, whose proliferation was set to 100%. (**c**) Curve shift experiment of the EGFR inhibitor gefitinib (red) and the HDAC inhibitor SAHA (blue) in a proliferation assay of the Hep-G2 cell line. (**d**) Calculation and visualization of synergy scores for drug combinations of Gefitinib and SAHA on Hep-G2 and Jurkat cells. Zero Interaction Potency (ZIP) synergy score calculated by Synergy Finder 2.0. A ZIP score from -10 to 10 describes that the interaction between two drugs is likely to be additive. Results are shown as means ± SEM of *n* ≥ 3 independent experiments. * *p* ≤ 0.05, ** *p* ≤ 0.005, *** *p* ≤ 0.0005, **** *p* ≤ 0.0001; linear regression.

The new chimeric compounds were investigated for their growth inhibitory effectiveness in human DU145 prostate cancer cells and human Hep-G2 hepatoblastoma cells (Table 1). Two compounds (3BrQuin-SAHA and 3ClQuin-SAHA) showed pronounced growth-inhibitory effects with IC_50_ values in the low micromolar range (~3–5 µM) in DU145 and Hep-G2 cells, that is, at concentrations far below those of the clinically relevant EGFR-inhibitor gefitinib, which was used as a reference for a single EGFR-inhibitor (IC_50_ of ~12 µM in DU145 cells and >18 µM in Hep-G2 cells). The IC_50_ value of SAHA, which served as clinically relevant reference for a single HDAC-inhibitor, amounted to ~3 µM in HepG2 cells, thus being in the range of those of 3BrQuin-SAHA and 3ClQuin-SAHA. In DU145 cells, SAHA was highly active, showing an IC_50_ value in the sub-micromolar range (~0.7 µM). Both 3BrQuin-SAHA and 3ClQuin-SAHA were further investigated for their time- and dose-dependent mode of action in DU145 and Hep-G2 cells (Figure 1a,b). Both 3BrQuin-SAHA and 3ClQuin-SAHA dose-dependently reduced the cell proliferation of DU145 and Hep-G2 cells significantly after only 24 h of treatment. The antiproliferative effects increased over time, and after 72 h, the maximum inhibition of prostate cancer DU145 cells amounted to >95% for both inhibitors. A comparable time- and dose-dependency was seen in hepatocellular Hep-G2 cancer cells with maximum inhibition of ~80% for 3BrQuin-SAHA and ~85% for 3ClQuin-SAHA after 72 h of treatment.

The combination of SAHA and Gefitinib did not lead to synergistic antiproliferative effects in Hep-G2 and Jurkat cells (Figure 1c). Further analysis of the combination treatment employing SynergyFinder2.0 software underlined this finding. The calculated synergy scores of 3.563 for Hep-G2 point towards an additive rather than a synergistic effect (Figure 1d). 

Unlike for solid tumors, single HDAC inhibitors such as SAHA or panobinostat are either already approved for the treatment of hematologic neoplasms such as T-cell leukemias/lymphomas or show promising pre-clinical data [10]. Nevertheless, the combination of kinase inhibition with HDAC inhibition has the potential to achieve significant improvements here, too. Hence, 3ClQuin-SAHA, which was the most active compound in the tested solid tumor cell models, was also selected for tests concerning its activity against a panel of lymphoma and leukemia cell lines (Jurkat, Hut78, SupT11, and SMZ1). The IC_50_ values of 3ClQuin-SAHA were in the low single-digit µM concentration range for all four cell lines (Table 1). Interestingly, 3ClQuin-SAHA was slightly more active than the SAHA, which served as a reference for a clinically approved HDAC-inhibitor. Likewise, the EGFR-inhibitor gefitinib showed a markedly less pronounced antiproliferative efficacy in Jurkat cells, and no appreciable effect in the other three T-cell lymphoma cell lines, as compared with 3ClQuin-SAHA. The novel chimeric inhibitor 3ClQuin-SAHA was shown to have pronounced antiproliferative potency also in hematologic tumor cell models. However, the enhanced antiproliferative effect of the 3ClQuin-SAHA in T-cell lymphoma cells may not be easily attributed to an additive or even synergistic effect of the HDAC-and EGFR-inhibiting pharmacophores of the compound, because additional FACS analyses on the expression of EGFR receptors in the T-cell lymphoma cell lines revealed that none of the four cell lines expressed EGFR receptors on their cell surface (Supplementary Figure S1). Not surprisingly, the combination treatment of Jurkat cells with SAHA and Gefitinib did not lead to synergistic or pronounced additive antiproliferative effects, but rather reflected the effect of SAHA (Figure 1d). Further investigations are needed to clarify the underlying mode of action of 3ClQuin-SAHA in lymphoma cells. Nevertheless, at this stage, the very fact that the novel chimeric inhibitors exert antiproliferative effects which are in the range of the clinically relevant monomodal HDAC-inhibitor SAHA underlines their broad efficacy. 

Hence, the novel chimeric inhibitor 3ClQuin-SAHA was shown to have superior antiproliferative potency also in hematologic tumor cell models. However, the enhanced antiproliferative effect of the 3ClQuin-SAHA in T-cell lymphoma cells may not be easily attributed to an additive or even synergistic effect of the HDAC-and EGFR-inhibiting pharmacophores of the compound, because additional FACS analyses on the expression of EGFR receptors in the T-cell lymphoma cell lines revealed that none of the four cell lines expressed EGFR receptors on their cell surface (Supplementary Figure S1). Thus, further investigations are needed to clarify the underlying mode of action of 3ClQuin-SAHA in lymphoma cells. Nevertheless, at this stage, the very fact that the novel chimeric inhibitors exceeded the antiproliferative effects of the clinically relevant monomodal HDAC-inhibitor SAHA underlines their broad efficacy. 

#### 2.2.2. Cytotoxicity of 3BrQuin-SAHA and 3ClQuin-SAHA 

**Figure 2:**
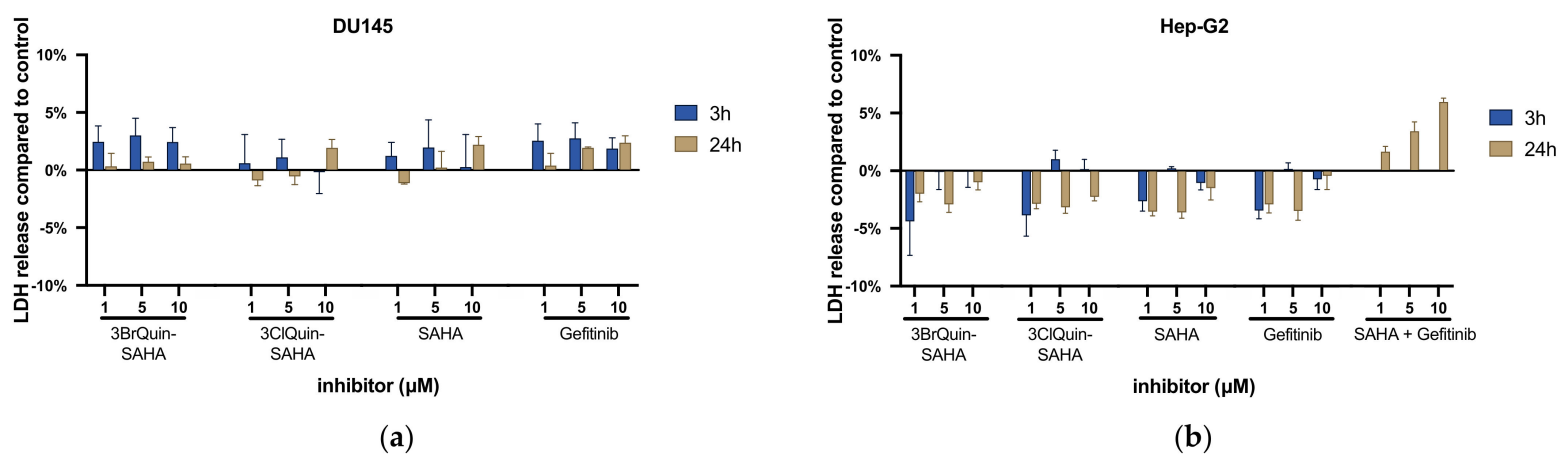
Unspecific cytotoxic effects of 3BrQuin-SAHA and 3ClQuin-SAHA in (**a**) prostate cancer and (**b**) hepatoma cells. LDH release of DU145 and Hep-G2 cells was measured after 3 and 24 h of incubation with 1, 5, or 10 µM of 3BrQuin-SAHA or 3Cl-Quin-SAHA, as well as SAHA and gefitinib alone or in combination on Hep-G2 cells. Results show changes in LDH release relative to untreated controls. Data are given as percentage changes relative to basal LDH release of controls. Means ± SEM of *n* = 3 independent experiments.

To check for cytotoxic effects possibly contributing to the antiproliferative effects of 3BrQuin-SAHA and 3ClQuin-SAHA treatment, the release of lactate dehydrogenase (LDH) from the cytosol into the supernatant of DU145 and Hep-G2 cell cultures was measured. Increased LDH release indicates necrotic cell death due to a treatment-induced damage of cell membranes [11]. However, neither 3BrQuin-SAHA nor 3ClQuin-SAHA induced significant increases in LDH release after 3 and 24 h of treatment with rising compound concentrations (1–10 µM), but rather showed a nonsignificant oscillation of ±5% around the basal LDH release of untreated controls (Figure 2). The data indicate that even at high concentrations, both compounds do not affect cell membrane integrity. Thus, an induction of immediate cytotoxicity is unlikely to account for the observed antiproliferative effects of the novel inhibitors. Moreover, as expected for SAHA and gefitinib, which were used as monomodal HDAC- and EGFR-inhibitor references, no relevant induction of necrosis was observed. Likewise, the combination of SAHA and gefitinib in Hep-G2 cells did not exhibit increased cytotoxicity either. (Figure 2b).

#### 2.2.3. EGFR Tyrosine Kinase Inhibition by 3BrQuin-SAHA and 3ClQuin-SAHA

**Figure 3:**
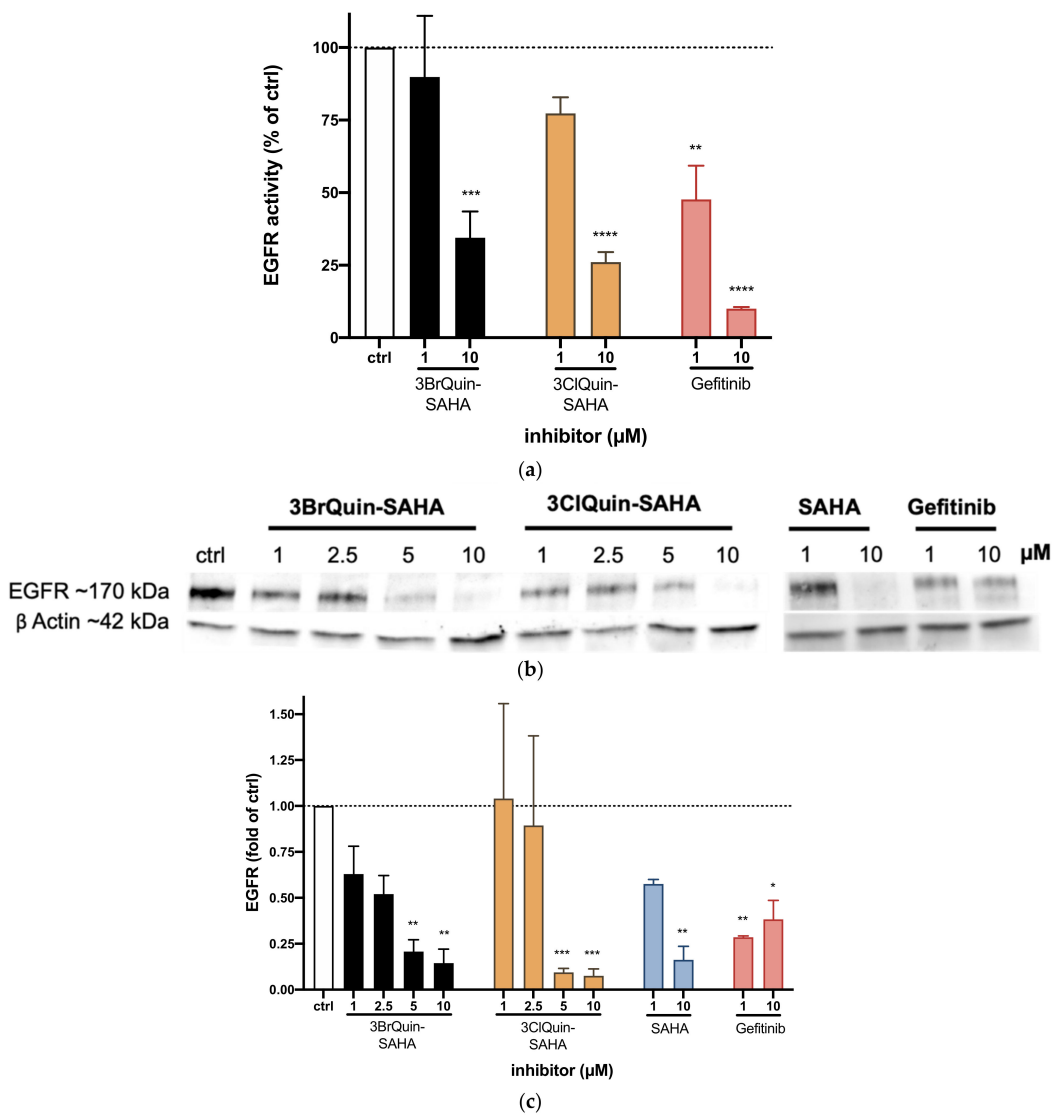
Inhibitory effects of 3BrQuin-SAHA and 3ClQuin-SAHA on EGFR tyrosine kinase activity and protein expression. (**a**) Dose-dependent inhibition of EGFR kinase activity by the novel chimeric inhibitors and gefitinib was determined in a cell-free EGFR-kinase assay. Means ± SEM of three independent experiments showing the percentage inhibition of treated vs. untreated controls whose kinase activity was set to 100%. (**b**) Representative Western blots of *n* = 3 independent experiments showing treatment induced changes in the expression of EGFR in DU145 cells after 24 h. ß-actin was used as loading control. (**c**) Out of these Western blots’ gray intensity, mean ± SEM of EGFRs’ specific bands were quantified and adjusted to protein-loading and normalized to untreated control. * *p* ≤ 0.05, ** *p* ≤ 0.005, *** *p* ≤ 0.0005, **** *p* ≤ 0.0001; two-way ANOVA Dunnett’s post hoc test.

Both 3BrQuin-SAHA and 3ClQuin-SAHA were tested for their EGFR tyrosine kinase inhibition by performing a luminescence-based, cell-free EGFR kinase activity assay. As shown in Figure 3a, a pronounced and dose-dependent inhibition of EGFR kinase activity of up to ~75% was observed upon treatment with the novel chimeric inhibitors. As expected, the clinically established EGFR-inhibitor gefitinib, used as a positive control, also showed a pronounced effectiveness and inhibited EGFR kinase activity up to ~85%. We also checked for cellular effects of the novel compounds by determination of changes in the protein level of the EGFR in DU145 prostate cancer cells. Western blots for the EGFR expression level of treated vs. nontreated DU145 cells revealed that 3BrQuin-SAHA and, even more so, 3ClQuin-SAHA led to a significant downregulation of EGFR protein (Figure 3b,c). The effect of both chimeric inhibitors was much more pronounced than that of gefitinib, and thus we wondered whether the HDAC-inhibitory moiety of the chimeric inhibitors may have an inhibitory effect on the EGFR protein expression. Indeed, treatment of DU145 cells with SAHA alone led to a pronounced and significant suppression in the expression of the EGFR that even exceeded that of gefitinib. Thus, we could show that the tyrphostin-like pharmacophore of the novel compounds is responsible for an effective inhibition of the EGFR-TK-activity and that the hydroxamate pharmacophore contributes to a significant suppression of the expression of the EGFR, which may lead to a synergistic or at least additional overall efficacy against EGFR-mediated cellular effects. 

#### 2.2.4. HDAC Inhibition by 3BrQuin-SAHA and 3ClQuin-SAHA

**Figure 4:**
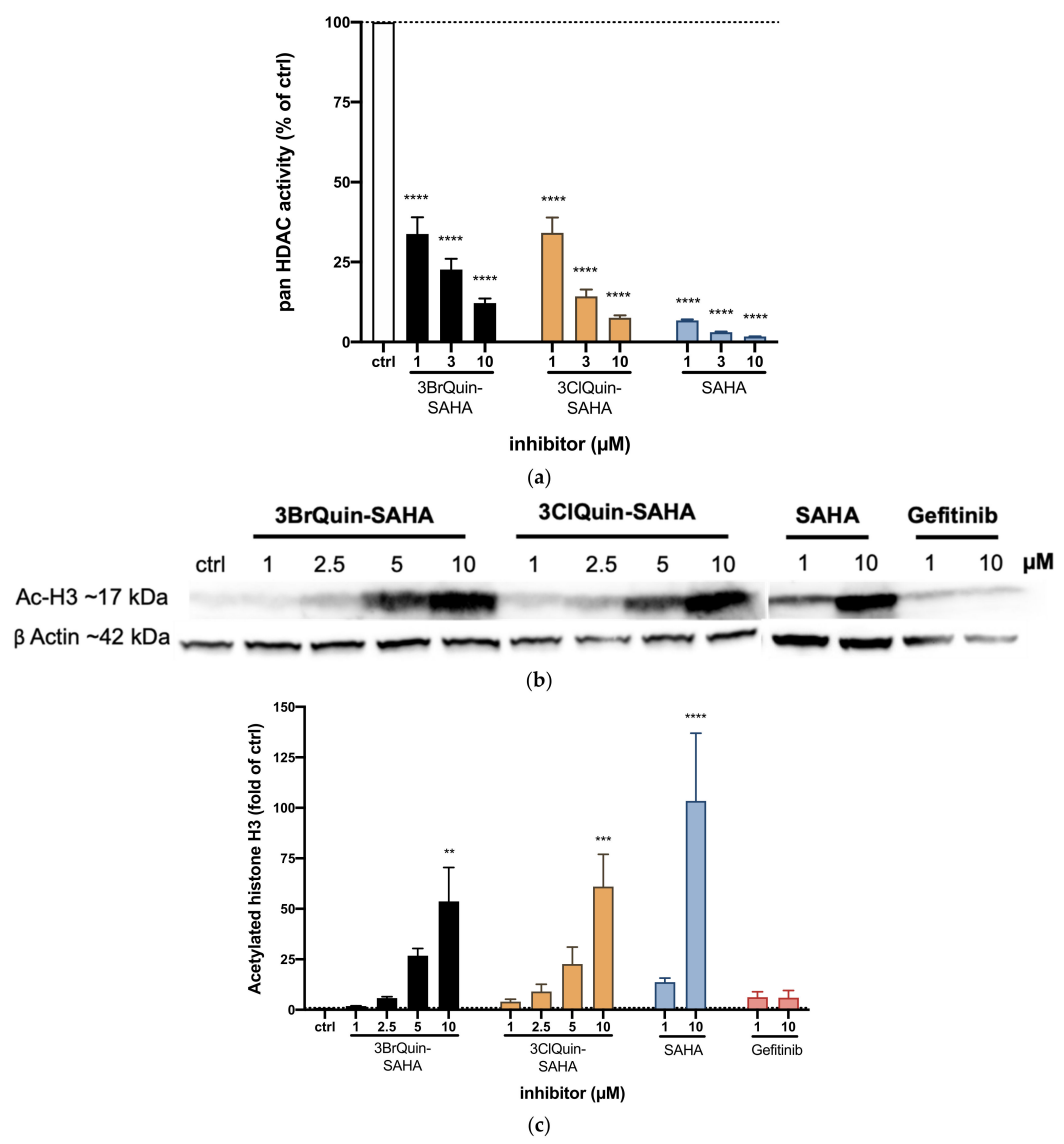

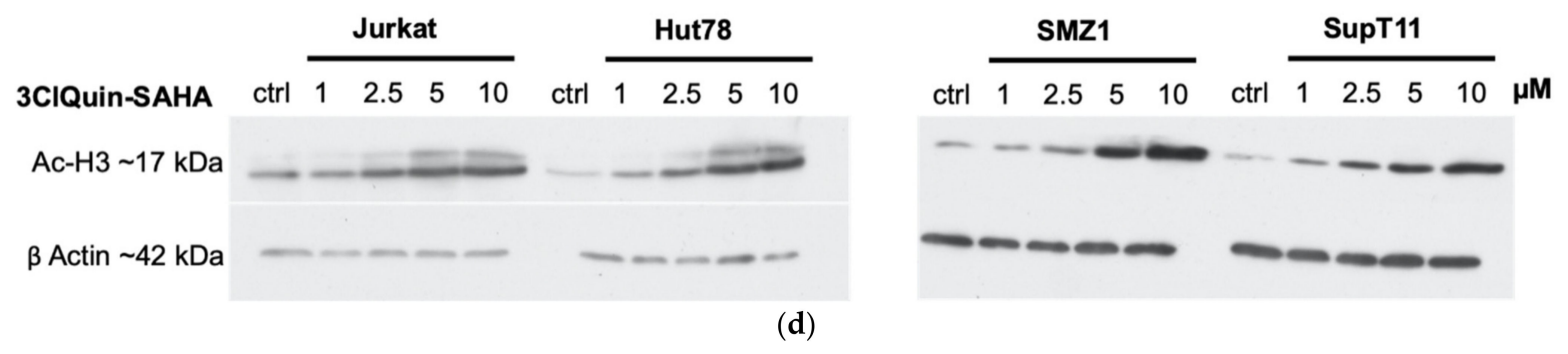
HDAC inhibitory effects of 3BrQuin-SAHA and 3ClQuin-SAHA. (**a**) HDAC activity was measured by luminescence display of the acetylation state of histones. HeLa nuclear cell extracts, used as source for histones, were preincubated with substrate and inhibitor. Results are given as relatives to controls, not preincubated with inhibitor, as means ± SEM of *n* = 3 independent experiment. (**b**) Representative Western blot out of *n* = 3 experiments, showing changes in the expression of acetylated histone H3 after 24 h treatment with compounds in DU145 cells. (**c**) Out of the DU145 Western blots’ gray intensity, mean ± SEM of acetylated histone-H3-specific bands were quantified, adjusted to protein-loading, and normalized to untreated control. (**d**) Western blots, showing changes in the expression of acetylated histone H3 after 24 h treatment with 3ClQuin-SAHA in Jurkat, Hut78, SupT11, andSMZ1 human T-cell leukemia/lymphoma cell lines. ** *p* ≤ 0.005, *** *p* ≤ 0.0005, **** *p* ≤ 0.0001; two-way ANOVA Dunnett’s post hoc test.

To assess the HDAC-inhibitory potency of the novel dual-mode hydroxamic acids, commercially available HeLa cell nuclear extracts, rich in HDAC activity and constituting a cell-free pan-HDAC enzyme profile, were treated with 3BrQuin-SAHA and 3ClQuin-SAHA (1–10 µM). Both compounds strongly inhibited the HDAC activity in a dose-dependent manner, leading to IC_50_ values in the sub-micromolar range with 302 nM (for 3BrQuin-SAHA) and 474 nM (for 3ClQuin-SAHA), respectively. Compared with the hydroxamic acid SAHA (IC_50_ = 20 nM), the mainstay of HDAC-targeting anticancer therapy, the pan-HDAC-inhibitory potency of the novel compounds is distinctly weaker, yet still in a clinically meaningful range (Figure 4a) [12].Their HDAC-inhibitory efficacy was further evaluated in prostate cancer cells by immunodetection of the increased portion of acetylated histone H3, which is part of the cellular nucleosome. Western blot analysis revealed a dose-dependent rise of H3 acetylation of DU145 cells upon treatment with 3BrQuin-SAHA and 3ClQuin-SAHA or SAHA as a consequence of the suppression of the histone deacetylating activity of HDACs (Figure 4b,c).

Increased acetylation of histone H3 upon treatment with 3ClQuin-SAHA was also seen in the four T-cell lymphoma cell lines (Figure 4d), confirming the HDAC-inhibitory activity of the compound also for the hematologic cancer cell models.

#### 2.2.5. Subtype Specific HDAC Inhibition of 3BrQuin-SAHA and 3ClQuin-SAHA

**Figure 5:**
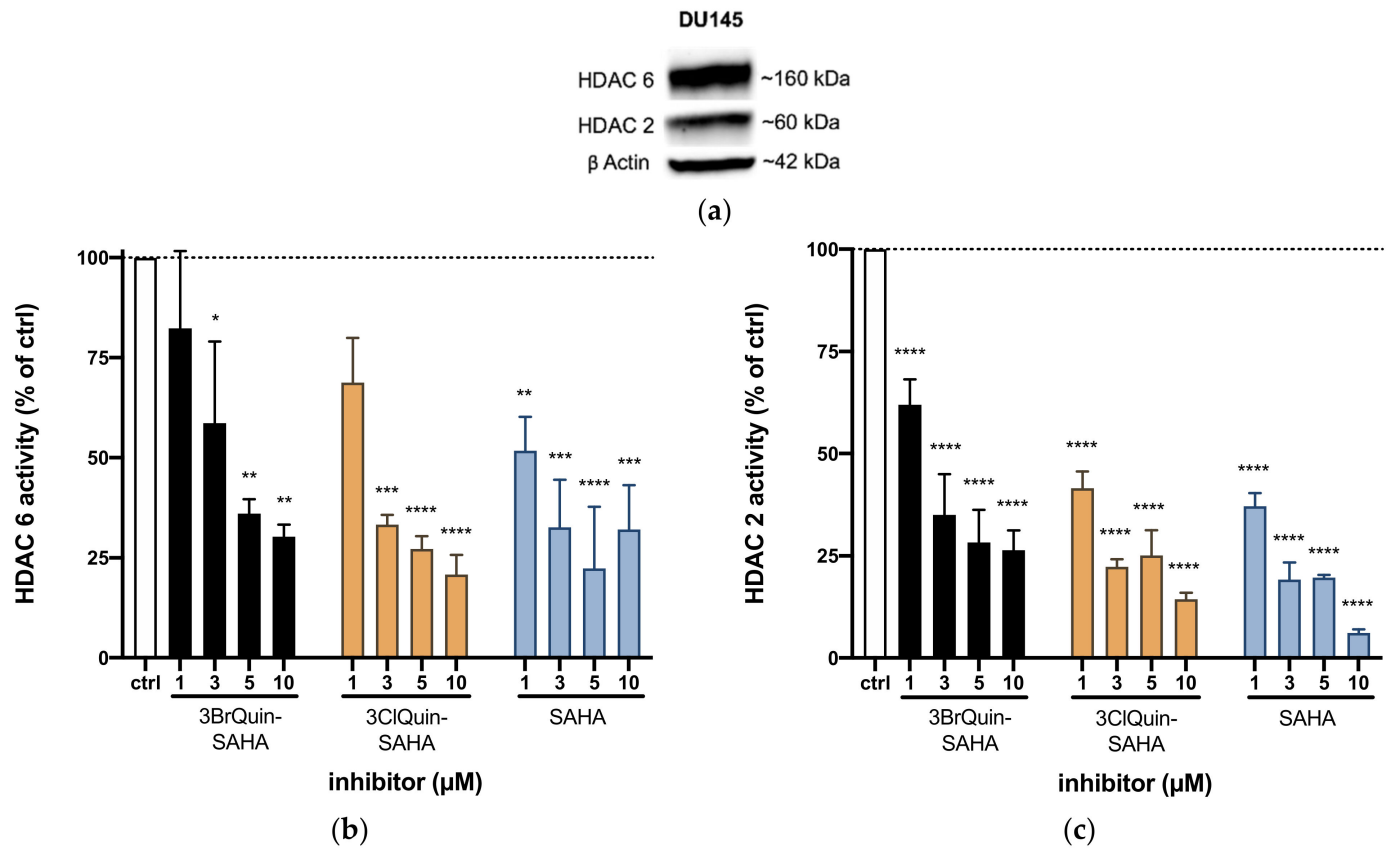
HDAC subtype specific inhibitory effects of 3BrQuin-SAHA and 3ClQuin-SAHA. (**a**) Western blot showing the expression levels of HDAC 2 and HDAC 6 in untreated DU145 cells (ctrl = control). (**b**) Dose-dependent inhibition of HDAC 6 and HDAC 2 (**c**) activity by 3BrQuin-SAHA, 3ClQuin-SAHA, or SAHA, respectively. Results, relative to untreated controls, as means ± SEM of *n* = 3 independent experiments. * *p* ≤ 0.05, ** *p* ≤ 0.005, *** *p* ≤ 0.0005, **** *p* ≤ 0.0001; two-way ANOVA Dunnett’s post hoc test.

Both 3BrQuin-SAHA and 3ClQuin-SAHA were also tested for HDAC-subtype-specific inhibition using recombinant HDAC 2 and 6 enzymes. Both HDACs showed high expression levels in untreated DU145 cells (Figure 5a). The activity of HDAC 6, interesting because of its cytosolic localization and chaperone modulating function, was shown to be strongly inhibited in a dose-dependent manner when treated with 1–10 µM of the novel chimeric compounds (Figure 5b) [13]. Similar effects were found for HDAC 2 (Figure 5c), a class 1 HDAC highly overexpressed in PCa and HCC cells [14,15]. 

#### 2.2.6. Induction of ROS and Apoptosis by 3BrQuin-SAHA and 3ClQuin-SAHA

**Figure 6:**
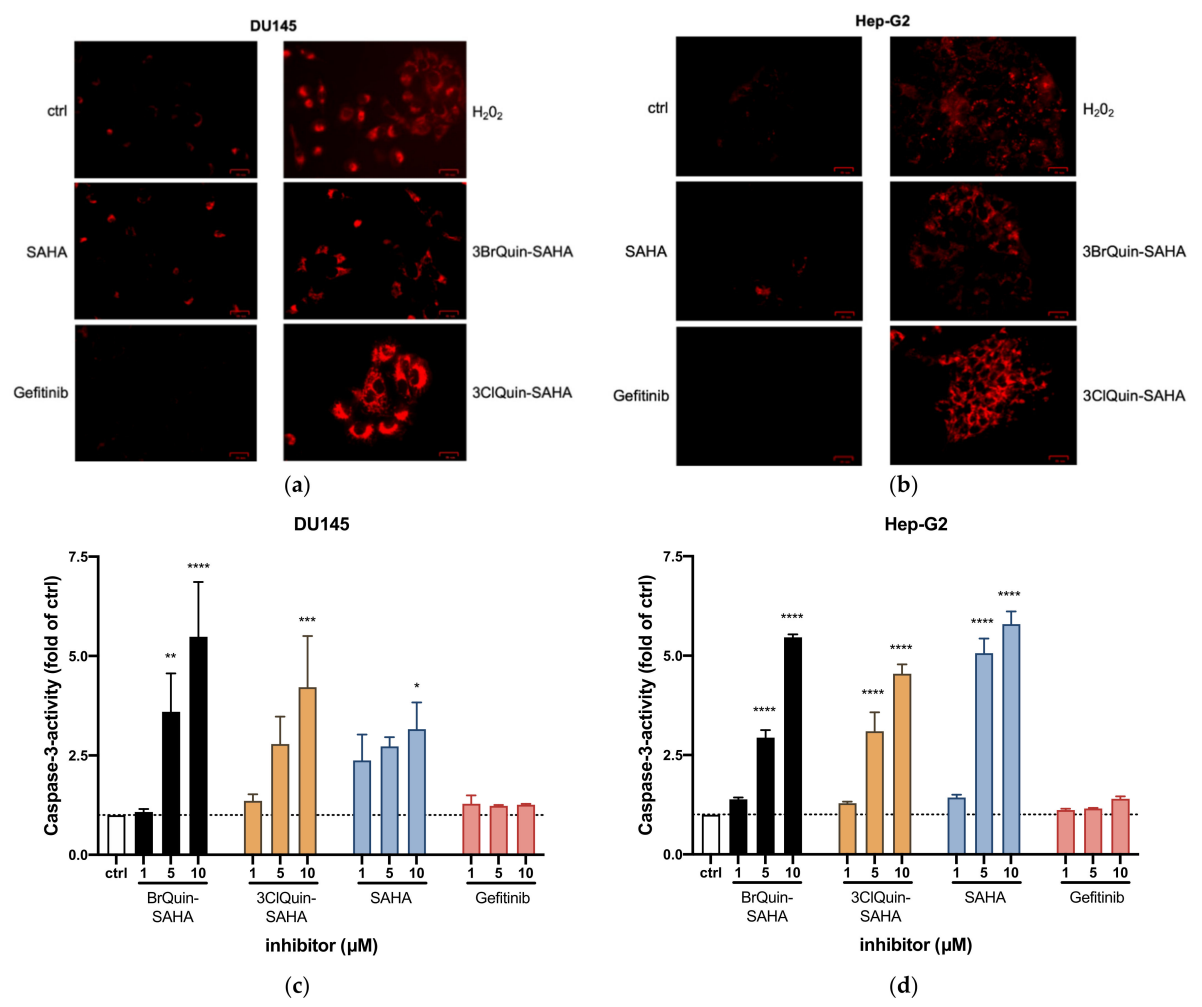

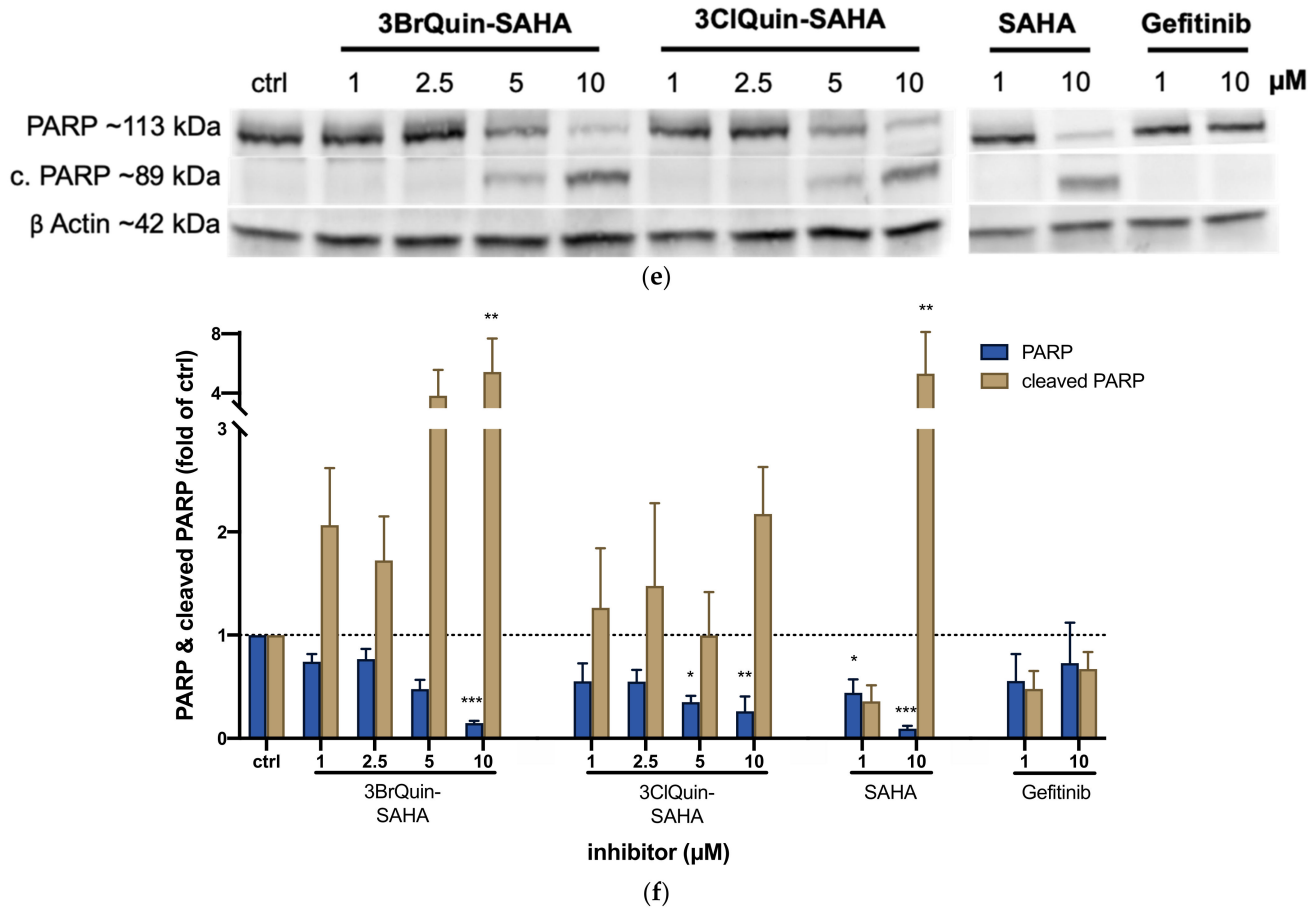
Apoptosis induction by 3BrQuin-SAHA and 3ClQuin-SAHA. ROS induction after 24 h of incubation with 10 µM 3BrQuin-SAHA, 3ClQuin-SAHA, SAHA, and gefitinib in DU145 (**a**) and for Hep-G2 cells (**b**). H_2_O_2_ (1.6 mM) was used as positive control. Representative findings out of *n* = 3 independent experiments. Caspase-3 induction in DU145 (**c**) or Hep-G2 cells (**d**) after 24 h treatment with 3BrQuin-SAHA, 3ClQuin-SAHA, SAHA, and gefitinib. Means ± SEM of *n* = 3 independent experiments. (**e**) Representative Western blot out of *n* = 3 experiments, showing changes in the expression PARP and PARP after cleavage in DU145 cells after 24 h treatment with compound. (**f**) Out of these Western blots’ gray intensities, PARP- and cleaved-PARP-specific bands were quantified, adjusted to protein-loading, and normalized to untreated controls. Data are given as means ± SEM of *n* = 3 independent experiments. * *p* ≤ 0.05, ** *p* ≤ 0.005, *** *p* ≤ 0.0005, **** *p* ≤ 0.0001; two-way ANOVA Dunnett’s post hoc test.

For a deeper insight into the molecular events underlying the mode of action of the novel chimeric inhibitors, we investigated their possible involvement in the induction of the formation of reactive oxygen species (ROS), since HDAC inhibition has already been shown to be linked to ROS induction in solid cancers, including prostate cancer [16]. Treatment of DU145 and Hep-G2 cells with 3BrQuin-SAHA and 3ClQuin-SAHA led to a pronounced time- and dose-dependent increase in cytosolic ROS after 24 h, as evidenced by fluorescence microscopy with the cytosol-specific ROS dye CellROX orange. Notably, a similar treatment with equimolar concentrations of SAHA or gefitinib elicited a much weaker (SAHA) or almost no (gefitinib) ROS increase in prostate and hepatocellular cancer cells (Figure 6a,b). 

ROS induction has been linked to the activation of caspases and may thus serve as a trigger of apoptosis [17]. To evaluate if apoptosis may play a role in the antiproliferative effects of the novel chimeric inhibitors, we checked them for the activation of the apoptosis-specific effector caspase-3. Upon treatment with 3BrQuin-SAHA and 3ClQuin-SAHA, a pronounced time- and dose-dependent caspase-3 activation became apparent. The onset of caspase-3 activation was visible after 6 h of treatment with 10 µM (Figure 6c,d). Treatment with SAHA elicited comparable caspase-3 inductions, whereas virtually no induction of caspase-3-activity was seen with gefitinib. Further Western blot analyses revealed a concomitant induction of apoptosis-specific cleavage of poly(ADP-ribose)-polymerase (PARP) (Figure 6e,f). PARP cleavage to its smaller ~85 kDa fragment is known to be caspase-3-driven and may be used as a marker for chemotherapy-induced apoptosis [18,19]. Our findings contribute to the assumption that apoptosis may play a prominent role for the effects that we observed upon treatment of Hep-G2 and DU145 cells with 3BrQuin-SAHA and 3ClQuin-SAHA. 

#### 2.2.7. In Vivo Assessment of Antiangiogenetic Effects of 3BrQuin-SAHA and 3ClQuin-SAHA on the CAM of Fertilized Chicken Eggs 

**Figure 7:**
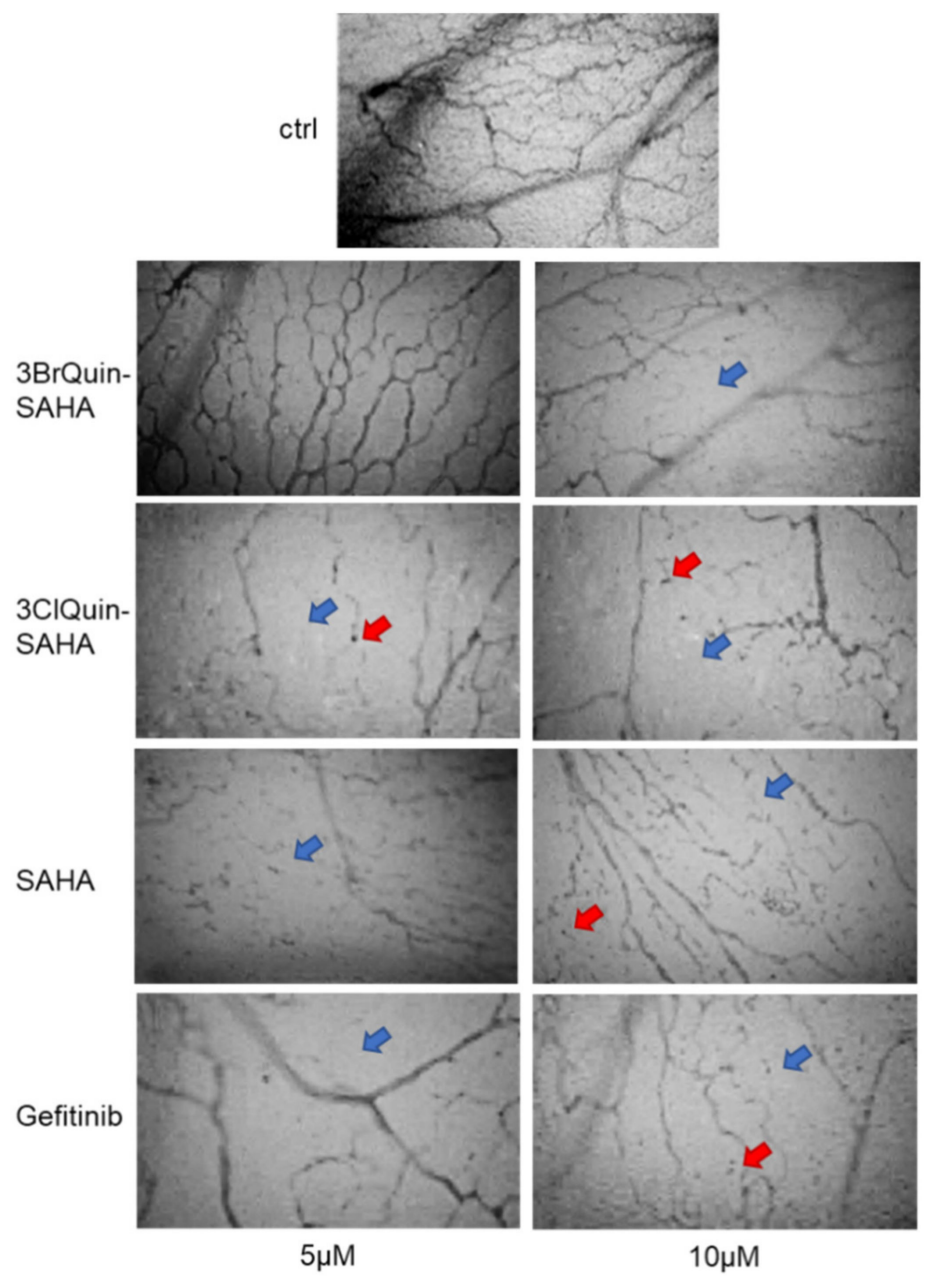
Antiangiogenic effects of 3BrQuin-SAHA and 3ClQuin-SAHA. Representative images from *n* = 3 preparations per condition of treatment-induced changes in the development and morphology of the vasculature of the chorioallantoic membrane (CAM) of fertilized chicken eggs after 72 h of topical treatment with 3BrQuin-SAHA, 3ClQuin-SAHA, SAHA, and gefitinib. Compared to untreated control CAMs, a dose-dependent reduction in the number (blue arrow) and altered morphology (red arrow) of CAM micro-vessels was observed. Most pronounced effects were seen in 3ClQuin-SAHA-treated CAMs.

Finally, the chimeric dual-mode inhibitors were screened for antiangiogenic effects in a systemic scenario by investigating treatment-induced changes in the vasculature of the chorioallantoic membrane (CAM) of fertilized chicken eggs (CAM assay) [20]. Qualitative comparisons between untreated (PBS) areas of the CAM to those treated with 3BrQuin-SAHA or 3ClQuin-SAHA showed a reduction of micro-vessels (Figure 7, blue arrows) after 72 h with the latter. Furthermore, respective single blood vessels displayed morphological irregularities such as blunted ends (Figure 7, red arrow). These features seemed to be more prominent in eggs treated with 3ClQuin-SAHA compared to those treated with 3BrQuin-SAHA. The monomodal reference compounds SAHA and gefitinib also showed antiangiogenic effects, which were, however, not as prominent as the ones caused by 3ClQuin-SAHA. Additionally, it is worth noting that there was no higher embryotic lethality observed in eggs treated with the compounds, which supports the notion from the LDH-release assays (Section 2.2.3; Figure 2) that the novel compounds do not exert general cytotoxic effects.

## 3. Discussion 

New tumor therapies for HCC and PCa are desperately needed due to poor treatment alternatives for patients with advanced tumor progression states. Resistance to first-line therapies is a common phenomenon and a major drawback for an efficient treatment [21,22]. As reported earlier, the overexpression of HDACs and EGFR is reported for tumors of different entities, including PCa and HCC [6,7,14,15].

In this study, we introduced new dual inhibitors combining tyrphostin AG1478- and indazole-derivates with a hydroxamic acid and addressing both EGFR and HDAC activities. 

Increased cell proliferation and compensatory inhibition of apoptosis are critical events that are required for the growth and development of cancer [23]. Targeting these key events is important for potent and specific anticancer therapies. We found that the two novel chimeric inhibitors 3BrQuin-SAHA and 3ClQuin-SAHA effectively inhibited the growth of PCa (DU145) and HCC (HepG2) cells at low micromolar concentrations, with IC_50_ (48 h) values between ~3–5 µM.

There are several studies which showed that targeting the HDAC activity in different cancer cells in combination with EGFR inhibition is a promising approach [24]. Bruzzese et al. demonstrated that the monomodal HDAC inhibitor SAHA in combination with the EGFR tyrosine kinase inhibitor gefitinib induced a synergistic inhibition of proliferation, migration, and invasion, as well as induction of apoptosis in squamous cell carcinoma of head and neck cells, including cells resistant to gefitinib [25]. Additional studies showed that the combination of these two drugs exhibited a high anticancer activity in various tumor entities [26,27]. The chimeric dual kinase/HDAC inhibitor CUDC-101 has already undergone clinical trials in patients with various solid tumors. The results were very promising, underlining the high relevance of this compound class [4].

To better understand the specific modes of action, the apoptosis induction via caspase-3-activity and PARP cleavage was investigated. It was found that 3BrQuin-SAHA, 3ClQuin-SAHA, as well as SAHA led to a prolonged activation of caspase-3 activity with a maximum of induction after 24 h of treatment. Concomitant to caspase-3 activation, PARP depletion due to its cleavage by caspase was observed in Western blot analyses. All these findings indicate that 3BrQuin-SAHA and 3ClQuin-SAHA are potent inducers of apoptosis in HCC and PCa cells. Their EGFR- and HDAC-inhibitory potency were proven in enzyme assays and Western blot analyses and confirmed by similar effects on purified HDAC2 and HDAC6 enzymes.

The expression of HDACs is upregulated in HCC and prostate cancer, and this upregulation is correlated with patient survival [14,15]. HDAC 2 expression was shown to be necessary for apoptosis prevention, mirrored by the finding that HDAC 2 inhibition increased apoptosis in colon carcinoma cells [28,29]. Quint et al. introduced HDAC 2 upregulation as a predictor for survival of HCC patients [30]. Several studies showed that HDAC inhibition by SAHA leads to higher apoptosis also in PCa tumor cells, independently of a specific HDAC subtype [31,32]. These findings are in line with our measurements showing high apoptosis rates in HCC and PCa cancer cells after treatment with SAHA but not with gefitinib. 

Interestingly, both new chimeric inhibitors as well as SAHA downregulated the expression of the EGFR in DU145 cells, indicating that the HDAC inhibitory moieties of the novel chimeric inhibitors may also affect the expression of EGFR. In conjunction with the findings on the regulation of EGFR activity in the cell-free EGFR kinase assays, our data indicate a dual activity of the novel compounds towards EGFR expression and downregulation of EGFR kinase activity. This would explain the enhanced overall anti-EGFR effect of the chimeric compounds compared to that of gefitinib alone. This could also be interesting for appraising expected/unexpected side effects, as well as therapeutic efficacy and therapy resistance [4].

In a first systemic evaluation employing CAM assays in which the vasculature of the chorioallantoic membrane of fertilized chicken eggs served as a substrate for the determination of antiangiogenic effects, we could finally show that 3BrQuin-SAHA and 3ClQuin-SAHA also exerted antiangiogenic effects on the vascular network of the CAM, while no appreciable cytotoxic side effects were observed, since the survival of the developing chicken embryos was not affected by the treatment. 

With this work, we shed first light on the modes of action of the two novel chimeric inhibitors 3ClQuin-SAHA and 3BrQuin-SAHA, combining HDAC- and EGFR-inhibiting moieties in one molecule. The compounds were found to encompass pronounced anti-proliferative, apoptosis-inducing, and anti-angiogenic effects in solid tumor cell models, warranting further studies to decipher in more depth the underlying modes of action and suitability of these promising representatives of the rapidly emerging class of chimeric inhibitors as candidates for future cancer treatment. 

## 4. Materials and Methods 

### 4.1. General Procedures and Materials

Chemicals and reagents were purchased from Sigma-Aldrich. Compounds 1a-c were prepared according to literature procedures [33,34]. The following instruments were used: determination of melting points (uncorrected), Gallenkamp; IR spectra generation, Perkin-Elmer Spectrum One FT-IR spectrophotometer with ATR sampling unit; obtaining nuclear magnetic resonance spectra (NMR), BRUKER Avance 300 spectrometer; chemical shifts are given in parts per million (δ) downfield from tetramethylsilane as internal standard; generating mass spectra (MS), Varian MAT 311A (EI); Thermo Fischer Scientific Q Exactive (ESI-HRMS); microanalyses, Elementar UNICUBE (Elementar Analysensysteme GmbH).

### 4.2. Synthesis of Novel Chimeric HDAC/EGFR Inhibitors 

**Ethyl-8-[4-(3-bromophenylamino)quinazolin-6-ylamino]-8-oxooctanoate (2a, 3BrQuin-SAHEt)**

4-(3-Bromophenylamino)-6-aminoquinazoline (80 mg, 0.254 mmol) was dissolved in dry CH_2_Cl_2,_ and ethyl hydrogen suberate (50 mg, 0.254 mmol), EDCI (75 mg, 0.39 mmol), DMAP (15 mg, 0.10 mmol), and triethyl amine (182 µL, 0.83 mmol) were added. After stirring at room temperature for 24 h, the solvent was evaporated, and the residue was purified by column chromatography (silica gel 60). Yield: 58 mg (0.12 mmol, 47%); *R*_f_ = 0.42 (ethyl acetate); colorless solid of m.p. 163–164 °C; υ_max_(ATR)/cm^−1^ 3286, 3099, 2931, 2852, 1733, 1667, 1627, 1593, 1572, 1526, 1476, 1418, 1392, 1381, 1357, 1327, 1290, 1242, 1226, 1176, 1132, 1090, 1072, 1062, 1030, 997, 974, 960, 949, 915, 869, 852, 843, 791, 777, 709, 681; ^1^H NMR (300 MHz, DMSO-d_6_) δ 1.17 (3 H, t, J = 7.1 Hz), 1.3–1.4 (4 H, m), 1.5–1.7 (4 H, m), 2.2–2.3 (2 H, m), 2.39 (2 H, t, J = 7.4 Hz), 4.05 (2 H, q, J = 7.1 Hz), 7.2–7.4 (2 H, m), 7.7–7.9 (3 H, m), 8.17 (1 H, s), 8.57 (1 H, s), 8.73 (1 H, s), 9.91 (1 H, s), 10.25 (1 H, s); ^13^C NMR (75.5 MHz, DMSO-d_6_) δ 14.1, 24.3, 24.8, 28.2, 28.3, 33.4, 36.1, 59.6, 111.6, 115.4, 120.8, 121.1, 124.3, 125.8, 127.1, 128.4, 130.3, 137.0, 141.2, 146.5, 152.9, 157.2, 171.4, 172.9; *m/z* (%) 500 (84) [M+], 498 (84) [M+], 455 (13), 453 (21), 315 (100), 234 (26), 83 (23), 69 (23), 55 (27), 41 (22).

***N*** **-[4-(3-Bromophenylamino)quinazolin-6-yl]-N-hydroxyoctanediamide (3a, 3BrQuin-SAHA)**

Ethyl-8-[4-(3-bromophenylamino)quinazolin-6-ylamino]-8-oxooctanoate (54 mg, 0.11 mmol) was dissolved in CH_2_Cl_2_/MeOH (9 mL, 1:2); then, hydroxylamine (50% in water, 0.5 mL, 15 mmol) and NaOH (200 mg, 5 mmol) were added, and the reaction mixture was stirred at room temperature for 1 h. The solvent was removed, and the residue was dissolved in water and adjusted to pH 8 with acetic acid. The aqueous phase was extracted with ethyl acetate (2 × 50 mL) with a small amount of MeOH, dried over Na_2_SO_4_ and concentrated in vacuum. The solid residue was recrystallized from CH_2_Cl_2_/*n*-hexane, washed with water in order to remove acetate impurities, and dried in vacuum. Yield: 58 mg (0.12 mmol, 47%); colorless solid of m.p. 241–242 °C; υ_max_(ATR)/cm^−1^ 3226, 2924, 2853, 1665, 1622, 1574, 1525, 1474, 1426, 1388, 1366, 1330, 1238, 1138, 1099, 1058, 981, 954, 925, 879, 840, 769, 723, 668; ^1^H NMR (300 MHz, DMSO-d_6_) δ 1.2–1.4 (4 H, m), 1.5–1.6 (2 H, m), 1.6–1.7 (2 H, m), 1.95 (2 H, t, J = 7.3 Hz), 2.38 (2 H, t, J = 7.3 Hz), 7.2–7.4 (2 H, m), 7.7–7.9 (3 H, m), 8.17 (1 H, s), 8.57 (1 H, s), 8.67 (1 H, s), 8.74 (1 H, s), 9.91 (1 H, s), 10.25 (1 H, s), 10.36 (1 H, s); ^13^C NMR (75.5 MHz, DMSO-d_6_) δ 24.9, 25.0, 28.4, 32.2, 36.2, 111.6, 115.5, 120.8, 121.1, 124.2, 125.8, 127.2, 128.4, 130.3, 137.0, 141.2, 152.9, 157.2, 171.4; *m/z* (ESI, %) 487.8 (83) [M+], 485.8 (85) [M+], 338.1 (100); Anal. calcd. (C_22_H_24_BrN_5_O_3_) C, 54.33; H, 4.97; N, 14.40; Found C, 54.10; H, 4.91; N, 14.29.

**Ethyl-8-[4-(3-chlorophenylamino)quinazolin-6-ylamino]-8-oxooctanoate (2b, 3ClQuin-SAHEt)**

4-(3-Chlorophenylamino)-6-aminoquinazoline (120 mg, 0.44 mmol) was dissolved in dry CH_2_Cl_2_ and ethyl hydrogen suberate (90 mg, 0.44 mmol), and EDCI (130 mg, 0.68 mmol), DMAP (26 mg, 0.174 mmol), and triethyl amine (315 µL, 1.43 mmol) were added. After stirring at room temperature for 24 h, the solvent was evaporated, and the residue was purified by column chromatography (silica gel 60). Yield: 76 mg (0.17 mmol, 39%); *R*_f_ = 0.38 (ethyl acetate); colorless solid of m.p. 159–160 °C; υ_max_(ATR)/cm^−1^ 3286, 3100, 2930, 2852, 1733, 1667, 1628, 1598, 1573, 1526, 1479, 1467, 1419, 1381, 1392, 1357, 1327, 1290, 1227, 1190, 1176, 1096, 1075, 1064, 1027, 999, 974, 951, 916, 880, 861, 843, 800, 791, 780, 710, 684; ^1^H NMR (300 MHz, DMSO-d_6_) δ 1.17 (3 H, t, J = 7.1 Hz), 1.3–1.4 (4 H, m), 1.5–1.7 (4 H, m), 2.2–2.3 (2 H, m), 2.39 (2 H, t, J = 7.4 Hz), 4.04 (2 H, q, J = 7.1 Hz), 7.1–7.2 (1 H, m), 7.3–7.4 (1 H, m), 7.7–7.9 (3 H, m), 8.04 (1 H, s), 8.57 (1 H, s), 8.72 (1 H, s), 9.89 (1 H, s), 10.22 (1 H, s); ^13^C NMR (75.5 MHz, DMSO-d_6_) δ 14.1, 24.3, 24.8, 28.2, 28.3, 33.4, 36.1, 59.6, 111.6, 115.4, 120.4, 121.4, 122.9, 127.1, 128.4, 129.9, 132.6, 137.0, 141.1, 146.5, 152.9, 157.2, 171.4, 172.8; *m/z* (%) 454 (93) [M+], 409 (21), 269 (100).

***N*** **-[4-(3-Chlorophenylamino)quinazolin-6-yl]-N-hydroxyoctanediamide (3b, 3ClQuin-SAHA)**

Ethyl-8-[4-(3-chlorophenylamino)quinazolin-6-ylamino]-8-oxooctanoate (74 mg, 0.16 mmol) was dissolved in CH_2_Cl_2_/MeOH (9 mL, 1:2). Hydroxylamine (50% in water, 0.5 mL, 15 mmol) and NaOH (200 mg, 5 mmol) were added, and the reaction mixture was stirred at room temperature for 1 h. The solvent was removed, and the residue was dissolved in water and adjusted to pH 8 with acetic acid. The aqueous phase was extracted with ethyl acetate (2 x 50 mL) with a small amount of MeOH, dried over Na_2_SO_4_ and concentrated in vacuum. The solid residue was recrystallized from CH_2_Cl_2_/*n*-hexane. Yield: 30 mg (0.068 mmol, 43%); colorless solid of m.p. 239–240 °C; υ_max_(ATR)/cm^−1^ 3229, 2924, 2854, 1665, 1621, 1600, 1574, 1521, 1487, 1427, 1394, 1366, 1332, 1266, 1239, 1137, 1105, 1057, 984, 958, 926, 878, 841, 800, 772, 694, 679; ^1^H NMR (300 MHz, DMSO-d_6_) δ 1.3–1.4 (4 H, m), 1.5–1.6 (2 H, m), 1.6–1.7 (2 H, m), 1.9–2.0 (2 H, m), 7.1–7.2 (1 H, m), 7.3–7.4 (1 H, m), 7.7–8.1 (4 H, m), 8.56 (1 H, s), 8.8–8.9 (1 H, m), 10.6–10.7 (1 H, m); ^13^C NMR (75.5 MHz, DMSO-d_6_) δ 24.5, 24.9, 28.4, 32.1, 36.1, 111.8, 115.5, 120.3, 121.3, 122.7, 127.0, 128.1, 129.9, 132.6, 137.3, 141.3, 146.3, 152.8, 157.3, 171.6; *m/z* (ESI, %) 443.9 (35) [M+], 441.9 (100) [M+], 428.9 (22), 426.9 (45), 282.1 (35); Anal. calcd. (C_22_H_24_ClN_5_O_3_) C, 59.79; H, 5.47; N, 15.85; Found C, 59.67; H, 5.53; N, 15.69.

**Ethyl-8-[4-(3-chloro-4-fluorophenylamino)quinazolin-6-ylamino]-8-oxooctanoate (2c, ClFQuin-SAHEt)**

4-(3-Chloro-4-fluorophenylamino)-6-aminoquinazoline (73 mg, 0.254 mmol) was dissolved in dry CH_2_Cl_2_ and ethyl hydrogen suberate (50 mg, 0.254 mmol). EDCI (75 mg, 0.39 mmol), DMAP (15 mg, 0.10 mmol), and triethyl amine (182 µL, 0.83 mmol) were added. After stirring at room temperature for 24 h, the solvent was evaporated, and the residue was purified by column chromatography (silica gel 60). Yield: 48 mg (0.102 mmol, 40%); *R*_f_ = 0.45 (ethyl acetate); yellow gum; υ_max_(ATR)/cm^−1^ 3335, 3144, 2935, 2861, 1733, 1680, 1617, 1572, 1549, 1498, 1426, 1386, 1365, 1332, 1300, 1242, 1215, 1182, 1129, 1094, 1070, 1058, 1033, 957, 932, 874, 830, 806, 779, 693, 657, 605; ^1^H NMR (300 MHz, CDCl_3_) δ 1.2–1.4 (7 H, m), 1.5–1.6 (2 H, m), 1.7–1.8 (2 H, m), 2.26 (2 H, t, J = 7.3 Hz), 2.39 (2 H, t, J = 7.3 Hz), 4.11 (2 H, q, J = 7.1 Hz), 7.0–7.1 (1 H, m), 7.3–7.5 (2 H, m), 7.7–7.9 (2 H, m), 8.27 (1 H, m), 8.42 (1 H, m), 8.64 (1 H, s), 8.85 (1 H, s); ^13^C NMR (75.5 MHz, DMSO-d_6_) δ 14.1, 24.5, 25.2, 28.6, 34.1, 37.3, 60.5, 110.7, 115.4, 116.2–116.5 (m), 120.7–120.9 (m), 121.7, 124.2, 126.1, 129.2, 135.0, 147.0, 153.1–156.4 (m), 153.9, 157.4, 172.3, 174.2; *m/z* (%) 474 (43) [M+], 472 (95) [M+], 427 (23), 288 (100).

***N*** **-[4-(3-Chloro-4-fluorophenylamino)quinazolin-6-yl]-N-hydroxyoctanediamide (3c, ClFQuin-SAHA)**

Ethyl-8-[4-(3-chloro-4-fluorophenylamino)quinazolin-6-ylamino]-8-oxooctanoate (48 mg, 0.102 mmol) was dissolved in CH_2_Cl_2_/MeOH (9 mL, 1:2). Hydroxylamine (50% in water, 0.5 mL, 15 mmol) and NaOH (200 mg, 5 mmol) were added, and the reaction mixture was stirred at room temperature for 1 h. The solvent was removed, the residue was dissolved in water, and adjusted to pH 8 with acetic acid. The aqueous phase was extracted with ethyl acetate, dried over Na_2_SO_4_, and concentrated in vacuum. The solid residue was recrystallized from CH_2_Cl_2_/*n*-hexane. Yield: 30 mg (0.065 mmol, 64%); colorless solid of m.p. >230 °C (dec.); υ_max_(ATR)/cm^−1^ 3224, 2924, 2852, 1664, 1626, 1572, 1503, 1412, 1333, 1240, 1213, 1138, 1088, 1043, 987, 960, 924, 877, 841, 812, 776, 647, 619; ^1^H NMR (300 MHz, DMSO-d_6_) δ 1.2–1.3 (4 H, m), 1.4–1.5 (2 H, m), 1.9–2.0 (2 H, m), 2.3–2.4 (2 H, m), 7.3–7.5 (1 H, m), 7.71 (1 H, d, J = 9.0 Hz), 8.0–8.1 (1 H, m), 8.2–8.4 (2 H, m), 8.54 (1 H, s), 9.1–9.2 (1 H, nr s), 11.1–11.2 (1 H, br s); ^13^C NMR (75.5 MHz, DMSO-d_6_) δ 24.9, 25.0, 28.3, 32.1, 36.1, 111.9, 115.6, 116.2–116.5 (m), 118.5–118.7 (m), 121.9, 123.0, 126.2, 127.8, 137.9, 145.8, 151.3, 162.6, 157.3, 171.6, 175.0; *m/z* (ESI, %) 461.9 (40) [M+], 459.9 (95) [M+], 316.2 (45), 288.2 (70); Anal. calcd. (C_22_H_23_ClFN_5_O_3_) C, 57.46; H, 5.04; N, 15.23; Found C, 57.38; H, 4.98; N, 15.17.

### 4.3. Biological Evaluations

#### 4.3.1. Cell Culture

Human hepatoma Hep-G2 cells (ATCC number: HB-8065) cells and human prostate cancer DU145 cells (ATCC number: HTB-81) were cultured in Roswell Park Memorial Institute 1640 Medium (RPMI) supplemented with 10% fetal bovine serum, 2 mM L-glutamine, 100 U/mL penicillin, and 100 mg/mL streptomycin (all from Gibco), and grown in an incubator (37 °C, 5% CO_2_, humidified atmosphere). Jurkat and SupT11 cell lines were purchased from DSMZ (Braunschweig, Germany). Hut78 cells were purchased from CLS Cell Lines Service GmbH (Eppelheim, Germany), and SMZ-1 cell line was provided by Dr. Raphael Koch from the University Göttingen. The four T-cell leukemia/lymphoma cell lines were cultured in RPMI-1640 medium including L-glutamine (Gibco) supplemented with 10% or 20% fetal bovine serum (Sigma-Aldrich, Darmstadt, Germany) and penicil-lin/streptomycin at 37 °C in a 5% CO_2_ incubator with 95% humidity.

#### 4.3.2. Compounds

Gefitinib and SAHA were purchased from Sigma-Aldrich, (Darmstadt Germany). Stock solutions (10 mM) were prepared in DMSO and stored at −20 °C. Working solutions were always freshly prepared before each experiment by dilution of stock solution with fresh cell culture medium. The final DMSO concentration in each experiment was below 0.25%. 

#### 4.3.3. Determination of Growth Inhibition

Treatment-induced changes in cell number were determined by crystal violet (N-hexamethylpararosaniline, Sigma Aldrich) staining as described before [35]. In brief, 1000 cells/well (DU145) or 1500 cells/well (Hep-G2) were seeded in 96-well plates and maintained for adherence in an incubator (37 °C, 5% CO2, humidified atmosphere) for 48 h prior to the beginning of the treatment. Subsequently, cells were treated with rising concentrations (0–10 µM) of the chimeric compounds, SAHA or gefitinib, respectively, for up to 72 h. 

Combinatorial drug treatment: HepG2 cells were treated with SAHA and Gefitinib for final concentrations of 0, 0.5, 1, 2.5, 5, and 10 μM for 48 h. Jurkat cells were treated with SAHA for final concentrations of 0, 1.5, 3, and 6 μM and 0, 5, 10, and 20 μM of Gefitinib for 48 h. 

Thereafter, the cells were fixed with 1% glutaraldehyde and stained with 0.1% crystal violet. The unbound dye was removed by rinsing with water. Bound crystal violet was solubilized with 0.2% Triton X-100 (Sigma-Aldrich). Light extinction of crystal violet, which increases linearly with the cell number, was analyzed at 570 nm using an ELISA-Reader (Dynex Technologies, Denkendorf, Germany). For T-cell leukemia/lymphoma cell lines IC_50_ values were determined using dual staining for Annexin-V and 7AAD via flow cytometry [10]. Time- and dose-dependent growth inhibition as well as IC_50_ values are given as means ± SEM of *n* ≥ 3 independent experiments performed in triplicates or more using Graph Pad Prism 8. Synergistic analysis was performed using SynergyFinder 2.0, utilizing the Zero Interaction Potency (ZIP) model [36]. A ZIP score less than -10 means the interaction between two drugs is likely to be antagonistic; from -10 to 10, the interaction between two drugs is likely to be additive; larger than 10, the interaction between two drugs is likely to be synergistic.

#### 4.3.4. Determination of Unspecific Cytotoxicity

To exclude unspecific cytotoxicity as the driving mode of action for antiproliferative effects, the release of lactate dehydrogenase (LDH) from DU145 and Hep-G2 cells was determined after 3 h and 24 h of treatment using the Cytotoxicity Detection Kit PLUS LDH (Roche Diagnostics GmbH, Mannheim, Germany). The assay was performed as described earlier [37]. Adherent non-treated cells, lysed with 2% Triton X-100 media for 10 min, served as reference values for maximum LDH release. Cytotoxicity was determined by subtracting the percentage of LDH release into the supernatant under control conditions of those from treated samples. Measurements were performed in duplicate in *n* = 3 independent experiments and mean percentage changes ± SEM as compared to controls are shown.

#### 4.3.5. EGFR Kinase Inhibition

To determine the EGFR tyrosine kinase-inhibitory potency of 3BrQuin-SAHA and 3ClQuin-SAHA, a cell-free EGFR kinase assay from Promega was employed (#3831) [38]. The clinically approved EGFR tyrosine kinase-inhibitor gefitinib (1 µM) was used as a positive control. Kinase reactions were carried out with EGFR (5 ng/μL), ATP (5 μΜ), and substrates (0.2 μg/μL) in kinase reaction buffer using kinase enzyme systems (Promega, Madison, WI, USA). Before the kinase reaction was started, enzyme and inhibitors were incubated at room temperature (22–25 °C) for 0.5 h. All kinase reactions were performed in 384-well plates with a volume of 5 μL and an incubation time of 1 h. To deplete the remaining ATP, 5 μL of ADP-Glo reagent (ADP-Glo kinase assay kit; Promega) was added to each well at RT for 40 min. Finally, 10 μL of kinase detection solution was added into each well of the 384-well plate. Luminescence was measured with a VarioSkan Flash 40053 microplate luminometer (Thermo Fisher Scientific, Waltham, Mass., USA) for 1 s. Measurements were performed in triplicate. Data of *n* = 3 independent experiments are given as means ± SEM of the percentage decrease in EGFR-TK activity, as compared to untreated controls, whose activity was set to 100%.

#### 4.3.6. Inhibition of HDAC Activity

The HDAC-inhibitory potential of 3ClQuin-SAHA and 3BrQuin-SAHA was deter-mined by using fluorogenic HDAC assay kits for the detection of pan-HDAC activity (Calbiochem, Merck Chemicals, Darmstadt, Germany) or the subtype-specific HDAC 2 and HDAC 6 activity (BPS Biosciences, San Diego, CA, USA). HDAC activity was measured according to the instructions of the supplier. SAHA (1–10 µM) served as positive control. Human HDAC enzymes derived from HeLa cell nuclear extracts (pan HDAC assay) or human recombinant HDAC-2 and HDAC-6 enzymes and adjacent fluorogenic HDAC substrates were used to determine HDAC activity levels. A 50 µL assay buffer containing 1 µg/µL bovine serum albumin, the human HDAC enzymes, 3ClQuin-SAHA (1–10 µM), 3BrQuin-SAHA (1–10 µM) or gefitinib (1–10 µM), and the corresponding HDAC substrates were added into a black 96-well assay plate. The reaction in each well was incubated at 37 °C for 30 min, followed by adding 50 µL HDAC developer reagent, and incubated at room temperature for an additional 15 min. Fluorescence intensity of the assay plates was measured on a Varioskan Flash fluorometer (Thermo Fisher Scientific, Waltham, MA, USA) using an excitation wavelength of 360 nm and an emission wavelength of 460 nm. 

#### 4.3.7. Measurement of Apoptosis-Specific Caspase-3 Activity

Changes in caspase-3 activity were measured by the cleavage of the fluorogenic substrate AC-DEVD-AMC (EMD Millipore, Billerica, MA, USA), as described previously [37]. After incubation with 3BrQuin-SAHA, 3ClQuin-SAHA, SAHA, or gefitinib (1–10 µM) for 6–48 h, the cells were harvested and lysed with lysis buffer. Subsequently, the lysates were incubated for 1 h at 37 °C with a substrate solution containing 20 μg/mL AC-DEVD-AMC, 20 mM HEPES, 10% glycerol, and 2 mM DTT at pH 7.5. Substrate cleavage was measured fluorometrically using a Varioskan Flash fluorometer (Thermo Fisher Scientific, Waltham, MA, USA; filter sets: ex 360/40 nm, em 460/10 nm).Three independent measurements were performed in triplicate, and data are given as the mean percentage increase ± SEM above control, which was set to 100%.

#### 4.3.8. Measurement of Reactive Oxygen Species (ROS)

The formation of cytosolic ROS in DU145 and Hep-G2 cells after treatment with 3ClQuin-SAHA, 3BrQuin-SAHA, SAHA or gefitinib (10 µM) was measured using the membrane permeable dye CellROX^®^ Orange (Thermo Fisher Scientific) which accumulates in the cytoplasm, exhibiting strong fluorescent signals at excitation/emission levels of 545nm/565nm upon oxidation [39]. Untreated cells incubated with 1.6 mM H_2_O_2_ for 30 min served as positive controls. CellROX^®^ Orange reagent (1 µM) was applied, when adding the test compounds. Formation of ROS was measured after 24 h using ZOE™ Fluorescent Cell Imager (Biorad, Munich, Germany). Independent experiments were performed in triplicate for each condition. 

#### 4.3.9. Western Blot 

Briefly, Western blots were performed as described earlier [40]. PCa cells were seeded in 100 mm petri dishes, grown to almost confluency, treated for 24 h, washed and frozen, followed by lysis with RIPA Buffer, added with one cOmplete^™^ Mini Protease Inhibitor tablet/10 mL (Roche), and quantification with Pierce™ BCA Protein Assay Kit (Thermo Fisher Scientific). Thereafter, protein levels were normalized to untreated controls, so protein-loading was equal to 20 µg/lane. Laemmli Buffer and β-mercaptoethanol were added before probes were desaturated at 96 °C for 10 min. First, 7.5% or 12% SDS gels (Biorad, Munich, Germany) were loaded with proteins and electrophoresis was done. Thereafter, proteins were transferred to activated polyvinylidene difluoride membranes (PVDF) by electroblotting. Finally, the membranes were blocked with 1% BSA and incubated with primary antibodies over night at 4 °C. The following antibodies directed against HDAC2 (5113S Cell signaling, 1:1000), HDAC6 (7558S Cell signaling, 1:1000), acetylated histone H3 (ab47915 abcam, 1:1000), EGFR (sc03 Santa Cruz Biotechnology, 1:500), poly-(ADP-ribose)-polymerase (PARP), and cleaved PARP (11835238 Roche, 1:1000), and β-actin (A5441 Sigma Aldrich, 1:2000) for standardization were used. Blots were washed three times with 1% TBS-Tween and incubated with anti-mouse or anti-rabbit peroxidase-coupled anti-IgG secondary antibodies (1:500–1:1000) at room temperature for 60 min. Subsequently, antibody bondage was illustrated using Clarity and Clarity Max ECL Western Blotting Substrates (Biorad, Munich, Germany) for detection and Celvin-S developer (Biostep, software SnapAndGo) for development. Independent blots of *n* = 3 experiments were generated, and the expression levels were determined by analyzing the bands with ImageJ and calculating the area under the curve (AUC) relative to its greyscale. Values were normalized to beta-actin expression, which served as a loading control, and compared to untreated control expressions, which was set 1. The mean ± SEM of the relative values to control are illustrated with Graph Pad Prism 8.

#### 4.3.10. In Vivo/Ovo Evaluation of Angiogenesis

Effects of 3BrQuin-SAHA and 3ClQuin-SAHA on angiogenesis were investigated on the CAM (chorioallantoic membrane) of fertilized chicken eggs [41]. Embryonic development is initiated by putting the eggs in an upright position into a humidified (>60%) incubator at 37.8 °C. At day 3 of egg development, a small 2 mm hole is pierced in the top of the eggshell, causing the developing CAM to detach from the eggshell and lower itself into the allantoic cavity. At day 10, the eggshell is further opened, and a silicone ring (1 cm Ø) is placed carefully on a region of interest of the CAM and allowed to attach for 6 h. Thereafter, 20 µL of inhibitor containing medium or PBS (control) is pipetted carefully into the ring. Changes in the angiogenic state of the CAM is then documented microscopically using a stereomicroscope equipped with a Kappa digital camera system (Distelkamp-Electronic, Kaiserslautern, Germany) by taking pictures every 24 h until the end of the experiment after 3 days of incubation. Experiments were performed with *n* = 3 eggs for each condition.

#### 4.3.11. Statistical Analysis

Statistical calculations were done with GraphPad Prism 8 (GraphPad Software, San Diego, CA, USA) using two-way ANOVA Dunnett’s post hoc test for statistical significance testing or linear regression. 

## Figures and Tables

**Table 1:** Growth-inhibitory activity expressed as IC_50_ (µM) of novel chimeric inhibitors in human prostate cancer (DU145), hepatoma (Hep-G2), and human T-cell lymphoma cell lines, as determined after 48 h. SAHA and gefitinib were used as clinically relevant references for monomodal HDAC- or EGFR-inhibition, respectively. All results are described as means ± SEM of *n* ≥ 3 (for sensitive) or *n* = 2 (for insensitive compounds) independent experiments.

Compounds	DU145	Hep-G2	Jurkat	Hut78	SupT11	SMZ1
3BrQuin-SAHA (3a)	3.53 ± 0.23	4.94 ± 0.38	n.d.	n.d.	n.d.	n.d.
3ClQuin-SAHA (3b)	3.23 ± 0.18	3.92 ± 0.25	1.40 ± 0.12	1.18 ± 0.22	6.22 ± 0.25	2.24 ± 0.17
ClFQuin-SAHA (3c)	5.57 ± 0.38	>10	n.d.	n.d.	n.d.	n.d.
SAHA	0.68 ± 0.04	3.22 ± 0.44	1.7 ± 0.17	5.07 ± 0.42	4.67 ± 0.31	2.87 ± 0.33
Gefitinib	11.88 ± 2.13	18.53 ± 1.78	10.95 ± 0.28	>20	>20	>20

## Data Availability

The data presented in this study are available on request from the corresponding author.

## Supplementary Materials

The following are available online at https://www.mdpi.com/article/10.3390/ijms22168432/s1, Figure S1: EGFR expression in T-cell lymphoma cell lines.

## Abbreviations

CAM	Chorioallantoic membrane
EGFR	Epidermal growth factor receptor
HCC	Hepatocellular carcinoma
HDAC	Histone deacetylase
LDH	Lactate dehydrogenase
PCa	Prostate cancer
ROS	Reactive oxygen species
