# Supplementary material for: Anticancer Activity and Mechanisms of Action of New Chimeric EGFR/HDAC-Inhibitors
Source: Int J Mol Sci. 2021 Aug 5;22(16):8432. doi: 10.3390/ijms22168432 (PMC8395095; doi:10.3390/ijms22168432)
Supplement: Supplementary file 1 [file ijms-22-08432-s001.zip › ijms-1305010-supplementary.pdf]

Supplementary Figure S1:

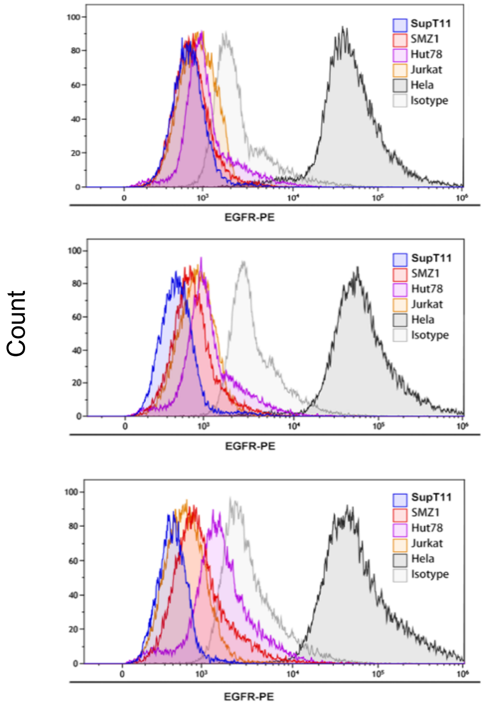

SF1: EGFR expression in T-cell lymphoma cell lines. Flow cytometry with PE-EGFR antibodies (anti-human EGFR antibody, Biolegend, # 352903, San Diego, USA) could not reveal a surface expression of the EGFR in the lymphoma cell lines SupT11, SMZ1, Hut78 and Jurkat, while HeLa cells, serving as EGFR-positive control cells, showed a strong signal. PE mouse IgG1,κ (Biolegend, # 4001139 was used a isotypic control antibody. The figure shows data from three individual repetitions of the experiment.
